# 基于科研能力提升的本科生实验课程设计：以“QuEChERS-气相色谱-质谱法测定土壤中嘧啶类杀菌剂实验”为例

**DOI:** 10.3724/SP.J.1123.2025.04022

**Published:** 2026-05-08

**Authors:** Fenglian LYU, Xiaoling SHAN, Ran ZHAO, Wei ZHENG

**Affiliations:** 1.西北农林科技大学资源环境学院，农业部西北植物营养与农业环境重点实验室，陕西 杨凌 712100; 1. Key Laboratory of Plant Nutrition and the Agro-environment in Northwest China，Ministry of Agriculture，College of Natural Resources and Environment，Northwest A&F University，Yangling 712100，China; 2.西北农林科技大学资源环境学院，资源与环境科学研究实验中心，陕西 杨凌 712100; 2. Experiment Center of Resources and Environmental Science Research，College of Natural Resources and Environment，Northwest A&F University，Yangling 712100，China

**Keywords:** 科研能力培养, 实验教学改革, 农药残留检测, QuEChERS, 气相色谱-质谱, scientific research ability training, experimental teaching reform, detection of pesticide residues, QuEChERS, gas chromatography-mass spectrometry

## Abstract

本科生实验课程是培养科研实践与创新能力的关键途径，传统实验教学“重流程、轻探究”的模式限制了学生科研思维与创新能力的发展，尤其在分析化学领域，GC-MS等技术教学多局限于验证性实验，难以满足需求。本文以“土壤中嘧啶类杀菌剂检测方法开发”为载体，构建了融合方法开发与问题探究的综合性实验教学方案，涵盖方法建立、技术优化、数据分析、拓展验证4个环节，采用小组合作与个人展示相结合的教学方式，引导学生优化QuEChERS-GC-MS相关参数，鼓励自主发现并解决问题。实验优化结果：1%乙酸乙腈溶液（20 mL）为最优提取溶剂；500 mg PSA+500 mg C18+500 mg GCB+1.2 g MgSO_4_为最佳净化组合；3种杀菌剂线性范围0.05~2.0 mg/L，相关系数≥0.995，检出限≤0.05 mg/kg，定量限≤0.167 mg/kg，适用于痕量检测。教学实践表明，该方案提升了学生的学习自主性与课堂互动，改善了实验报告质量，为后续科研实验奠定了基础，对高校相关专业实验教学改革具有重要推广价值。

## Abstract (en)

Undergraduate experimental courses are a key way to cultivate students’ scientific research practice and innovation abilities. The traditional experimental teaching mode of “emphasizing process and neglecting inquiry” limits the development of students’ scientific research thinking and innovation ability. Especially in the field of analytical chemistry， technical teaching involving techniques such as GC-MS is limited to confirmatory experiments， which is difficult to meet practical needs. In this paper， a comprehensive experimental teaching scheme integrating method development and problem exploration is constructed based on the development of detection methods for pyrimidine fungicides in soil. It covers four links： method establishment， technology optimization， data analysis and extended verification. The teaching method of group cooperation and personal display is adopted to guide students to optimize the relevant parameters of QuEChERS-GC-MS method and encourage them to independently discover and solve problems independently. The results showed that 1% acetic acid acetonitrile （20 mL） was the optimal extraction solvent； 500 mg PSA+500 mg C18+500 mg GCB+1.2 g MgSO_4_ was the best purification combination； the linear range of the three fungicides was 0.05-2.0 mg/L， the correlation coefficient was ≥0.995， the LOD was≤0.05 mg/kg and the LOQ was ≤0.167 mg/kg， which was suitable for trace detection. Teaching practice shows that the scheme improves students’ learning initiative and classroom interaction， enhances the quality of experimental reports， lays a foundation for subsequent scientific research experiments， and has important promotion value for the experimental teaching reform of related majors in colleges and universities.

高等教育背景下，实验教学是培养学生实践能力和科研素养的关键环节。然而，当前高校实验教学普遍存在“重流程、轻探究”的问题^［1］^。学生在实验操作过程中仅限于按照教师安排的固定步骤操作，缺乏对实验原理和实验方法的深入理解和探究。这种教学模式虽然能够保证学生掌握基本的实验操作技能，但却难以激发学生对实验原理的深度思考和对实验方法的创新性改进，难以满足创新性人才培养需求。在分析化学领域，气相色谱-质谱法（GC-MS）作为复杂基质痕量分析的核心技术^［2，3］^，其教学多局限于固定方法的验证性实验。然而，随着分析化学领域对科研能力要求的不断提高，传统的实验教学模式难以满足学生对科研能力提升的需求，亟待改革与创新。

近年来，随着分析化学技术的快速发展，QuEChERS技术因其快速、简便、成本低等优点，已逐步替代传统固相萃取法（SPE）、液液萃取法（LLE）等技术^［4-7］^，成为复杂基质样品前处理的首选方法^［8，9］^。QuEChERS技术结合GC-MS在环境监测、食品安全、药物分析等领域得到了广泛应用^［10，11］^。已有研究证实，QuEChERS结合GC-MS对果蔬中多农药残留检测效果显著^［12，13］^，但针对土壤基质中嘧啶类杀菌剂的应用仍缺乏系统性研究，尤其对极性嘧啶类化合物的净化策略及仪器参数优化尚未充分探讨。此外，将这些先进技术融入本科实验教学的实践仍相对较少。

基于此，本研究通过创新实验教学方案，有效解决了传统实验课程中存在的问题。以“QuEChERS-气相色谱-质谱法测定土壤中嘧啶类杀菌剂实验”为例，将前沿技术和方法开发过程转化为实验教学模块，通过方法建立-方法优化-拓展研究的螺旋式训练，提升学生解决分析化学实际问题的综合能力。

## 1 实验部分

### 1.1 仪器与试剂

Trace 1610-TSQ 9610型气相色谱-质谱仪（美国赛默飞世尔科技有限公司），配备先进的电子电离（AEI）源；HC-3018R型高速冷冻离心机（安徽中科中佳科学仪器有限公司）；MX-F型涡旋混合仪（美国赛洛捷克仪器有限公司）。

3种嘧啶类杀菌剂标准品：嘧霉胺（pyrimethanil）、嘧菌环胺（cyprodinil）、嘧菌腙（ferimzone），纯度≥98%，购自上海安谱实验科技有限公司。净化试剂：*N*-丙基乙二胺（PSA）、十八烷基硅胶（C18）、石墨化炭黑（GCB）购自上海安谱实验科技有限公司。丙酮（C_3_H_6_O）、乙腈（C_2_H_3_N）、乙酸（CH_3_COOH）、氯化钠（NaCl）、无水硫酸镁（MgSO₄）购自上海阿拉丁试剂公司。

### 1.2 标准溶液的配制

标准储备液：精确称量标准品，用丙酮充分溶解得到3种目标物浓度为100 mg/L的标准储备液。混合标准储备液：分别准确移取3种标准储备液各1 mL，移入10 mL容量瓶，以丙酮定容，涡旋混匀后备用。取适量的混合标准储备液，用丙酮溶液按照逐级稀释法配制质量浓度为0.05、0.10、0.5、1.0、1.5、2.0 mg/L的混合标准溶液。

### 1.3 样品采集与前处理

空白土壤：取一定量的无污染土壤（校园土），置于烘箱内150 ℃烘烤6 h，以除去水分及其他挥发性干扰物，处理后置于干燥器内冷却备用，该样品用于实验条件优化测试分析。

样品土壤：学生按分组自行采集土壤样品5份，并进行充分混匀代表该农田的一个样品，样品采集完成后立即带回实验室放置于阴凉干燥处，风干后进行研磨过筛，备用，该样品用于后续的拓展性实验测试分析。

取2.5 g风干空白土壤，加入1%乙酸乙腈溶液20 mL，涡旋1 min，离心5 min（6 000 r/min）。取上清液10 mL，加入500 mg PSA+500 mg C18+500 mg GCB+1.2 g MgSO_4_净化剂组合，涡旋1 min，冷冻离心5 min（8 000 r/min），取上清液5 mL，氮吹仪吹至近干，加入1 mL丙酮，过0.22 μm滤膜备用，上机测定。

### 1.4 分析条件

色谱柱：TG-5 SIMLS石英毛细管色谱柱（30 m×0.25 mm×0.25 μm，美国赛默飞世尔科技有限公司）；进样方式：不分流进样；进样量：1 μL；柱流速：1.2 mL/min；程序升温：起始温度75 ℃，保持2 min，以20 ℃/min升至200 ℃，保持6 min，再以15 ℃/min升至280 ℃，保持5 min；进样口温度：240 ℃。AEI源：70 eV；离子源温度：230 ℃，传输线温度：250 ℃；溶剂延迟3 min；扫描模式：全扫描（Full Scan）和选择离子扫描（SIM），全扫描确定目标物质保留时间、定量离子和定性离子后，进行SIM模式扫描。3种嘧啶类杀菌剂在SIM模式下的质谱参数见表1。

**表1:** 3种嘧啶类杀菌剂的质谱参数

Compound	Retention time/min	Quantitative ion （*m/z*）	Qualitative ions （*m/z*）	Collision energy/eV
Pyrimethanil	10.56	198.1	157.6， 117.9	30
Cyprodinil	14.44	224.1	196.9， 208.0	20
Ferimzone	15.30	344.1	156.0， 171.9	34

## 2 结果与讨论

### 2.1 质谱参数的优化

采用TG-5 SIMLS石英毛细管色谱柱对3种嘧啶类杀菌剂进行分离，分别通过Full Scan和SIM模式进行定性和定量测定，选择丰度高、干扰低的特征离子作为定量离子，丰度较高、干扰较低的2个特征离子作为定性离子。本实验根据质谱参数对混合标准溶液进行上机测定，获得3种嘧啶类杀菌剂的总离子流色谱图（图1）。

**图1:**
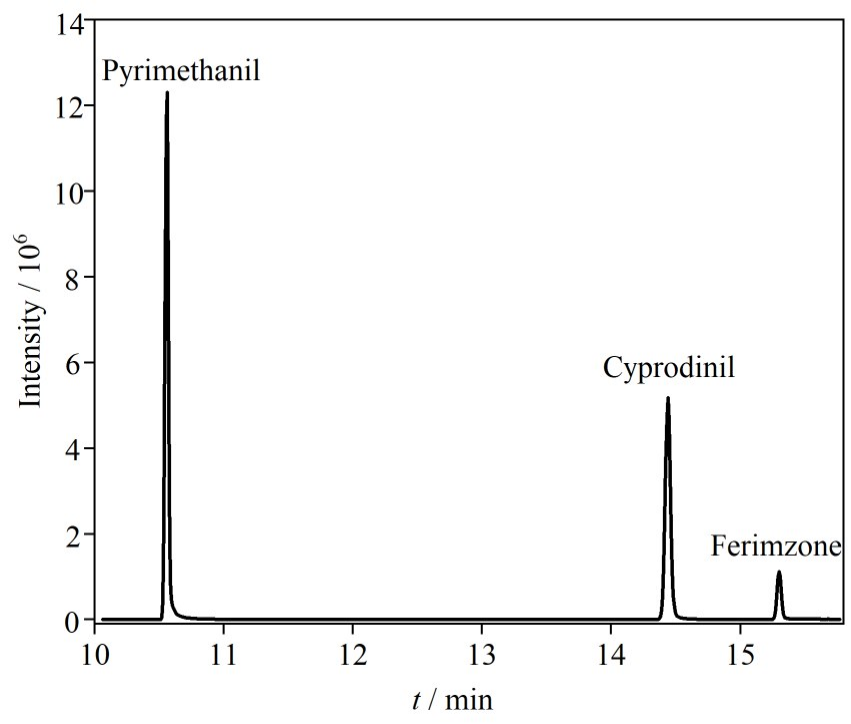
3种嘧啶类杀菌剂的总离子流色谱图

### 2.2 前处理条件的优化

#### 2.2.1 提取溶剂的优化

基于溶剂的溶解能力、提取效率、兼容性和实验优化条件的综合考虑，本实验选用丙酮、乙腈、1%乙酸乙腈溶液3种常用的提取溶剂进行盐析萃取/分配，探究不同的提取溶剂对基质提取效率的差异，并针对多种提取溶剂对土壤样品中嘧啶类杀菌剂的提取效果进行评价，选出最优的提取溶剂；其次，对溶剂的使用剂量（10、20、30 mL）进行分析并结合实验结果，综合考虑提取效率和成本选择出合适的提取溶剂用量。结果发现，当采用1%乙酸乙腈溶液作为提取溶剂，使用量为20 mL时，嘧菌环胺、嘧霉胺和嘧菌腙的回收率均高于丙酮和乙腈。推测原因可能是嘧菌环胺、嘧霉胺和嘧菌腙3种杀菌剂均呈弱碱性，在乙酸溶液的弱酸性条件下更稳定，回收率也有所提高，结果见图2。因此，本研究选用1%乙酸乙腈溶液20 mL作为优化提取溶剂。

**图2:**
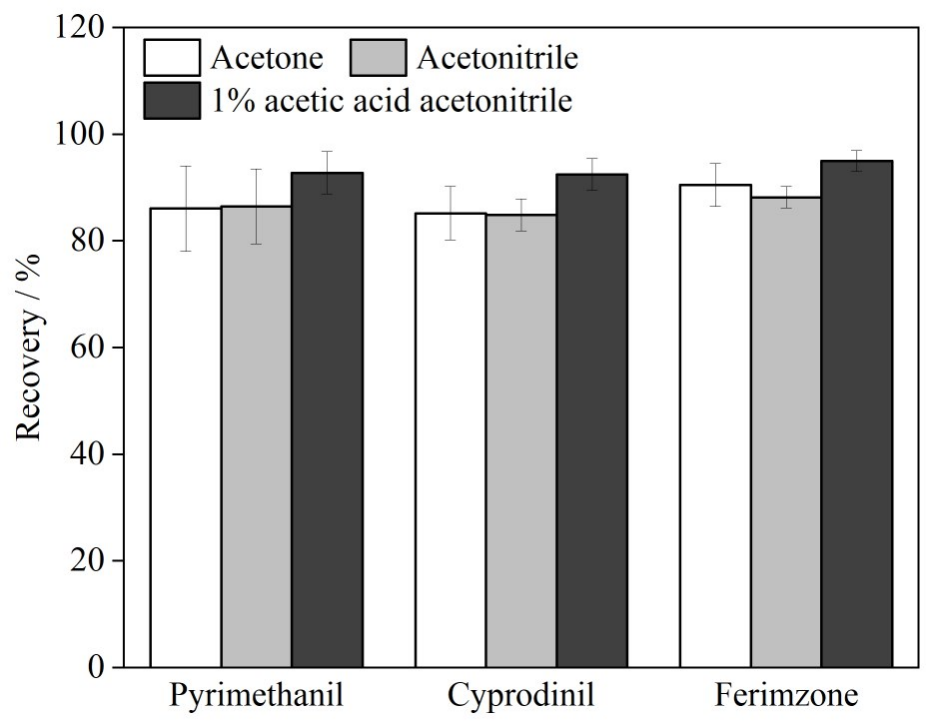
不同萃取溶剂对回收率的影响（*n*=3）

#### 2.2.2 净化条件的优化

由于土壤成分复杂，本实验选用不同组合及配比的PSA、GCB、C18、MgSO_4_为考察因素并设置3种类型的净化剂组合（表2）。结果发现，采用500 mg PSA+500 mg C18+500 mg GCB+1.2 g MgSO_4_的净化组合时，测定得到的目标物干扰峰最少。说明不同净化剂的配比会影响其对杂质的吸附能力。另外，本实验中最优净化剂组合“500 mg PSA+500 mg C18+500 mg GCB+1.2 g MgSO_4_”可能是由比例和净化填料的总重量共同作用的结果。特别是在土壤成分复杂的情况下，一方面适宜配比的净化剂组合可能更有效地去除特定杂质，同时保留目标化合物；另一方面，增加净化剂的总重量能有效去除杂质，并减少基质效应。

**表2:** 不同配比的净化剂具体用量

Type	PSA/mg	C18/mg	GCB/mg	MgSO_4_ /g
A	300	300	50	1.2
B	500	500	500	1.2
C	100	100	0	1.2

### 2.3 线性范围与检出限

在优化测试条件下，对3种嘧啶类杀菌剂的混合标准工作溶液（质量浓度范围0.05~2.0 mg/L）进行测定，以3种杀菌剂的质量浓度为横坐标（*x*），对应峰面积响应值为纵坐标（*y*）绘制标准曲线。3种嘧啶类杀菌剂在0.05~2.0 mg/L范围内表现出良好的线性关系，线性相关系数（*R* ^2^）≥0.995。以信噪比（*S/N*）=3和*S/N*=10确定检出限（LOD）和定量限（LOQ），分别为0.005~0.050 mg/kg和0.017~0.167 mg/kg，满足农药残留检测的定量要求（见表3）。

**表3:** 3种嘧啶类杀菌剂的线性方程、相关系数、线性范围、检出限及定量限

Germicide	Linear equation	*R* ^2^	Linear range/（mg/L）	LOD/（mg/kg）	LOQ/（mg/kg）
Pyrimethanil	*y*=1.854×10^5^ *x*-1.118×10^4^	0.995	0.05-2.0	0.050	0.167
Cyprodinil	*y*=1.252×10^5^ *x*-8.078×10^3^	0.997	0.05-2.0	0.005	0.017
Ferimzone	*y*=1.080×10^4^ *x*-1.149×10^3^	0.998	0.05-2.0	0.010	0.033

*y*： peak area； *x*： mass concentration， mg/L.

## 3 教学实践与改革成效

### 3.1 教学实践

#### 3.1.1 实验教学设计

通过系统化的实验教学，能有效衔接理论学习和实践应用，为提升学生科研能力提供保障。本实验课程设计方案主要由实验理论部分、实验材料准备、实验实践及应用三部分组成。（1）课程理论部分设计。课程理论部分主要利用课堂时间完成，包括设备操作、方法探索、实验设计、成果输出等。具体内容及预期目标见表4。（2）实验材料准备。在课程开始前，准备实验用设备并确保其正常运行、实验用试剂、采集空白样品和实验样品等。（3）实验实践及应用。为了确保实验课程中理论与实践的高效结合，该部分内容主要包括样品前处理过程、仪器设备原理与样品上机测定、数据综合分析、实验报告撰写、拓展实验项目研究等。通过系统性和规范化的实践训练，可以提升学生的动手能力和科研素养，进一步培养解决问题和锻炼独立科研的能力。

**表4:** 实验理论与预期目标

Theoretical teaching	Training content	Training objectives
Equipment operation	principles of GC-MS and scanning mode setting	specifications for precision instrument operation
Method exploration	comparative experiment of extraction solvents	ability in variable control and comparative data analysis
Experimental design	optimization of purification effect	application of experimental design methods
Result output	writing experimental reports in standard format	skills in scientific paper writing and chart presentation

#### 3.1.2 实验课程安排

课前准备：课前发布学习任务，激发学生的主动探索。①文献研读任务：要求学生在课程开始前，查阅与本实验紧密相关的文献资料，精准把握实验背景、原理和潜在应用价值。学生需要提交一份简短的文献综述报告，提炼关键信息要点。②预实验规划：学生依据实验目的，自主构思初步实验方案，涵盖实验流程、预期结果以及可能遇到的难题等。教师在课前对学生的预实验方案进行审核，给出针对性建议。③问题征集：积极鼓励学生在课前梳理出与实验相关的问题，这些问题将在课堂上集中讨论。借此契机，充分调动学生的思考与探索积极性。

实验课程安排及学生分组：实验理论讲解、实验实践部分利用课堂时间完成，样品采集与保存、实验材料准备在课程开始前利用课外时间完成。具体实验教学时间安排（5学时）：实验理论部分1.5学时；实验前处理操作部分2学时；仪器设备使用1.5学时。数据处理、结果分析及实验报告在课外时间完成。实验采用小组协作模式开展，班级人数一般为25~32人，可分为3组，每组约9人，并配备组长1名。每组聚焦一项具体的实验任务，小组内成员分工明确，锻炼学生的协作配合与沟通交流能力（表5）。

**表5:** 小组内成员具体分工

Group	Member	Extraction agent type	Extraction agent dosage/mL	Purification agent type	Sample serial number
Group 1	1	acetone	10	A	1
	2	acetone	10	B	2
	3	acetone	10	C	3
	4	acetone	20	A	4
	5	acetone	20	B	5
	6	acetone	20	C	6
	7	acetone	30	A	7
	8	acetone	30	B	8
	9	acetone	30	C	9
Group 2	1	acetonitrile	10	A	10
	2	acetonitrile	10	B	11
	3	acetonitrile	10	C	12
	4	acetonitrile	20	A	13
	5	acetonitrile	20	B	14
	6	acetonitrile	20	C	15
	7	acetonitrile	30	A	16
	8	acetonitrile	30	B	17
	9	acetonitrile	30	C	18
Group 3	1	1% acetic acid acetonitrile	10	A	19
	2	1% acetic acid acetonitrile	10	B	20
	3	1% acetic acid acetonitrile	10	C	21
	4	1% acetic acid acetonitrile	20	A	22
	5	1% acetic acid acetonitrile	20	B	23
	6	1% acetic acid acetonitrile	20	C	24
	7	1% acetic acid acetonitrile	30	A	25
	8	1% acetic acid acetonitrile	30	B	26
	9	1% acetic acid acetonitrile	30	C	27

课堂讨论：结合实验过程，在问题关键环节进行讨论。（1）质谱参数优化环节，讨论：①3种杀菌剂的母离子和保留时间；②确定3种杀菌剂的定性和定量离子。通过课堂问题式讨论，可以检查学生课前预习情况，以及锻炼学生对专业问题的分析和解决能力。最终，探讨出优化后的质谱参数，用于后续定量分析实验过程。（2）提取溶剂优化环节，该部分涉及提取剂类型及用量的比较，要求班级学生全员全程参与，包括实验前处理过程、实验上机测定及实验结果综合分析。实验结束后将测定结果进行组内和组间共享探讨出最优的提取剂类型和用量，旨在培养学生间的团队协作能力、科研开放思维，增强团队意识。同时，根据测定结果，讨论：①样品回收率如何计算？②1%乙酸乙腈溶液20 mL作为提取剂时，回收率高的原因？③该提取溶剂类型和用量是否适用于所有类型农药残留检测？通过问题分析，培养学生的综合分析能力，并为学生进一步拓展实验研究项目提供方向。（3）净化条件优化环节，根据组内数据综合分析，发现“500 mg PSA+500 mg C18+500 mg GCB+1.2 g MgSO_4_”的净化剂组合目标物的干扰峰最少，可能是由比例和净化填料的总重量共同作用的结果。因此，为了明确原因，后续实验方案将进一步设置：①固定净化填料的总重量，改变配比的实验，通过实验结果判断不同配比对结果的影响；②净化填料固定比例，改变总重量的实验，观察实验结果的变化。通过该实验设计和分析方法，可以更准确地判断最优结果是由比例影响还是由净化剂总重量导致的，从而为实验条件优化提供科学依据。（4）标准曲线的绘制，该环节是理工科实践能力培养的基础，要求组内成员过程性全部参与，为拓展性实验研究奠定良好的基础。

#### 3.1.3 成果汇报与拓展性实验

成果展示：小组成果汇报。在实验结束后，每个小组面向全班展示实验成果，包括实验数据、结果图表以及实验结论。要求学生条理清晰地阐述实验全过程以及结果分析，借此锻炼语言表达和逻辑思维分析能力。另外，在小组汇报过程中，其他小组需对汇报小组的成果进行评价，并提出问题或个人见解。这种互评和提问环节，能够有效激活学生的批判性思维，促使学生从多元视角剖析问题。最后，教师在学生充分讨论后，对实验结果进行总结与点评，并给出改进建议。教师点评应聚焦实验结果本身，同时注重学生思维过程与问题解决方法。

拓展性实验：样品土壤检测。学有余力或兴趣使然或有毕业设计相关要求的学生可以利用组内及组间采集的样品土壤进行自主实验实践。采用优化后的前处理方法对样品土壤进行处理，并测定3种嘧啶类杀菌剂含量。在该过程中，教师进行适度引导，学生独立完成实验操作流程，让学生在实践中自行探寻问题解决路径，积累实践经验。该部分主要满足学生在科研上的兴趣，提供实践锻炼的机会，这不仅有利于学生科研思维的提升，也有利于培养学生的创新素养。

A： 300 mg PSA+300 mg C18+50 mg GCB+1.2 g MgSO_4_； B： 500 mg PSA+500 mg C18+500 mg GCB+1.2 g MgSO_4_； C： 100 mg PSA+100 mg C18+1.2 g MgSO_4_.

### 3.2 课程改革成效

该实验课程从2021级环境工程专业本科生开始实施，效果显著，主要体现在以下几个方面。

（1）提高学生学习的自主性。本实验课程通过明确分工，使每个学生都承担独特的任务，确保每个学生的参与度。学生的失误或不积极将直接影响整个小组甚至班级的数据完整性，这种机制极大地提高了学生学习的积极性和主动性。学生不再被动地接受知识，而是主动探索和解决问题，自主学习能力显著提升。

（2）增强课堂互动与师生交流。本课程通过随堂互动和问题讨论，鼓励学生主动思考和提问。在实验操作过程中，学生不再依赖组长或个别同学，而是通过团队合作解决问题。这种模式不仅提高了学生的实践能力，还增加了师生之间的互动交流，教师能够及时了解学生的学习进度和困难，提供针对性指导意见。

（3）提升实验报告质量。由于每个学生获得的实验数据不同，学生需要基于自身数据进行分析并通过组内和组间数据的共享，形成综合性结论。这种多样化的数据处理方式，促使学生更加注重实验数据的准确性和分析深度，从而提升实验报告的质量；学生在撰写实验报告时，不再局限于模板化的描述，而是能够结合实际数据进行深入分析和讨论。

（4）为后续科研实验奠定基础。根据院内教师反馈，通过该实验课程学习的学生，在毕业论文设计、实验操作及科研论文撰写方面有了明显的提升。学生不仅掌握了实验技能，还培养了科研思维和创新能力，为后续开展科研实验奠定了良好的基础。

## 4 结论与展望

本实验建立的QuEChERS-GC-MS方法适用于土壤中痕量嘧啶类杀菌剂的快速筛查与定量分析。通过优化前处理方法与仪器测定参数，有效降低了基质干扰，为其他结构类似农药多残留监测提供了可靠的技术支撑。此外，基于科研能力提升的教学模式，不仅提高了学生的自主学习能力和实践能力，还增强了团队协作能力和创新思维能力，为培养更多理论与实践并存的优秀人才夯实基础。

未来，我们将根据新技术发展趋势和方向，不断拓展实验实践类项目，为学生发展提供实践平台。同时，我们将结合现代信息技术，开发在线实验教学资源，提高教学效率和质量。

## References

[1] Yang H F， Wang Y Y， Hu J J， et al . University Education， 2024， 94（16）： 52

[2] Fan S L， Liu Y M， Yan F P . Guangzhou Chemistry， 2023， 51（8）： 242

[3] Zhang R Z， Xu H Z . Shandong Chemical Industry， 2024， 53（7）： 226

[4] Liu Y N， Jin X Z， Gao H Y， et al . Journal of Analytical Science， 2021， 37（2）： 151

[5] Saito-shida S， Nagata M， Nemoto S， et al . Food Addit Contam： Part A， 2021， 38（1）： 125 10.1080/19440049.2020.1846082 33232630

[6] Rakesh K G， Zareen S K， Kaushik B， et al . J AOAC Int， 2020， 103（1）： 55 31466554

[7] Tomasz K， Alicja N， Stanislaw S， et al . J Chromatogr A， 2016， 1435： 100 26830634

[8] Li W W， Wang Y， Wang S N， et al . Journal of Food Safety & Quality， 2020， 11（6）： 1852

[9] Huang X H， Huang Y Y， Gao W， et al . Chinese Journal of Chromatography， 2025， 43（4）： 388 40133205 10.3724/SP.J.1123.2024.06010 PMC11966371

[10] Liu X C， Huang L， Zhu M M， et al . Journal of Zhejiang Agricultural Science， 2017， 58（3）： 472

[11] Liu R Q， Wang Q J， Cao Q H， et al . Guizhou Agricultural Sciences， 2016， 44（6）： 67

[12] Yi C S， Liu R， Wu Z P， et al . Chinese Journal of Chromatography， 2024， 42（3）： 282 38503705 10.3724/SP.J.1123.2023.07018 PMC10951808

[13] Li Y M， Zhou Y P， Pan W L， et al . Agrochemicals， 2024， 63（1）： 39

